# Enhancing the effect of repetitive I-wave paired-pulse TMS (iTMS) by adjusting for the individual I-wave periodicity

**DOI:** 10.1186/1471-2202-12-45

**Published:** 2011-05-18

**Authors:** Sebastian Sewerin, Marco Taubert, Henning Vollmann, Virginia Conde, Arno Villringer, Patrick Ragert

**Affiliations:** 1 Max Planck Institute for Human Cognitive and Brain Sciences, Department of Neurology, D-04103 Leipzig, Germany

**Keywords:** transcranial magnetic stimulation (TMS), I-wave periodicity, motor evoked potential (MEP), primary motor cortex (M1), corticospinal excitability, paired-pulse stimulation

## Abstract

**Background:**

Repeated application of paired-pulse TMS over the primary motor cortex (M1) in human subjects with an inter-pulse interval (IPI) of 1.5 ms (iTMS_1.5 ms_) has been shown to significantly increase paired-pulse MEP (ppMEP) amplitudes during the stimulation period and increased single-pulse MEP amplitudes for up to 10 minutes after termination of iTMS.

**Results:**

Here we show in a cross-over design that a modified version of the iTMS_1.5 ms _protocol with an I-wave periodicity adjusted to the individual I1-peak wave latency (iTMS_adj_) resulted in a stronger effect on ppMEPs relative to iTMS_1.5 ms_.

**Conclusions:**

Based on these findings, our results indicate that the efficiency of iTMS strongly depends on the individual choice of the IPI and that parameter optimization of the conventional iTMS_1.5 ms _protocol might improve the outcome of this novel non-invasive brain stimulation technique.

## Background

Over the past decade, various non-invasive brain stimulation protocols such as repetitive transcranial magnetic stimulation (rTMS) or transcranial direct current stimulation (tDCS) have been introduced to either up- or down-regulate cortical excitability within the stimulated cortical area of human subjects (for review see [1,2]). For example, it has been shown that application of high-frequency rTMS protocols (≥ 5 Hz) leads to an increase of corticospinal excitability beyond the time of stimulation while low-frequency rTMS protocols (≤ 1 Hz) generally decreases it [2]. The outcome of non-invasive brain stimulation protocols, however, critically depends on many parameters such as intensity, frequency and duration of stimulation (for review see [2]). Single-pulse TMS applied over the primary motor cortex (M1) in healthy human subjects elicits a brief train of descending volleys at a periodicity of approximately 1.5 ms which are assumed to result from an indirect activation of corticospinal neurons via cortical interneurons (I-wave activation) [3,4]. More recently, repeated application of paired-pulse TMS over M1 for 30 minutes with an inter-pulse interval (IPI) of 1.5 ms (iTMS_1.5 ms_) has been shown to (a) significantly increase paired-pulse MEP (ppMEP) amplitudes during the stimulation period and (b) increase single-pulse MEP amplitudes for up to 10 minutes after termination of iTMS_1.5 ms _[5]. In a further study by the same group, Cash et al. demonstrated the effect of iTMS on individual I-wave components non-invasively [6]. They found that 15 min of iTMS_1.5 ms _with an IPI of 1.5 ms increased all three I-wave (I1-I3) peaks suggesting that iTMS_1.5 ms _increased the efficacy of synaptic events by a direct modification of descending volleys. The decisive parameter of this procedure, i.e. the value of the IPI, meets the assumed 1.5 ms average of I-wave latency in human subjects and thus may induce a "Hebbian-like" effect of plasticity. Following this reasoning and given recent evidence that the individual I-wave latency in human subjects shows remarkable inter-subject variability [6,7] we hypothesized that the effect of iTMS_1.5 ms _could be increased by individually adjusting the IPI.

Thus, in this study we tested whether an iTMS protocol with an IPI adjusted to the individual I-wave periodicity (iTMS_adj_) has a stronger effect on paired-pulse MEP (ppMEP) amplitudes during stimulation as compared to the conventional iTMS_1.5 ms _protocol [4-6,8,9].

## Results

**Figure 1:**
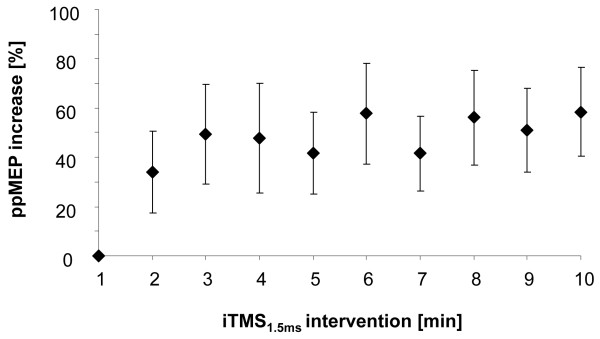
**Increased ppMEPs during iTMS_1.5 ms_**. Group mean ppMEP increase [%] during 10 minutes of conventional iTMS_1.5 ms _normalized to the first minute of stimulation (n = 16). Group mean data is presented as mean ± s.e.m. Note that there was a significant increase in ppMEP facilitation during intervention. For details see text.

All subjects tolerated both interventions without reporting any unexpected discomfort and there was no adverse event during the study procedure. Ten minutes of conventional iTMS_1.5 ms _over the left M1 resulted in a significant increase in ppMEPs during intervention (ANOVA_RM _with factor TIME (1-10 min): F_(9,135) _= 3.680; p = 0.005; see Figure 1). After 10 minutes of iTMS_1.5 ms_, ppMEPs were increased by 58.41 ± 18.03% (mean ± s.e.m., Figure 1) relative to baseline. However, 5 out of 16 subjects tested showed an overall decrease in ppMEPs during intervention (-22.11 ± 6.24%).

**Figure 2:**
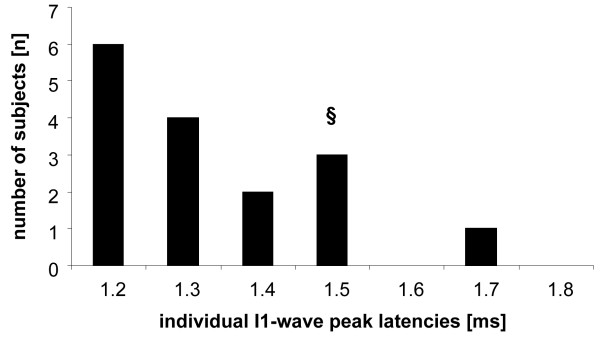
**Distribution of individual I1-wave peak latencies [ms] in all subjects tested (n = 16)**. § indicates that three subjects with an individual I1-wave peak latency of 1.5 ms were excluded from further analysis in order to compare the efficacy of iTMS_adj _relative to iTMS_1.5 ms_.

Measuring the individual I1-wave peak latency for each subject revealed a mean peak ranging around 1.3 ms (see Figure 2). Interestingly, only 3 out of 16 subjects showed an I1-wave peak latency around 1.5 ms. These subjects were excluded from further analysis since the aim of the present study was to compare the outcomes of iTMS_1.5 ms _and iTMS_adj _where the IPI should be different from 1.5 ms. For the distribution of individual I1-wave peak latencies see Figure 2.

**Figure 3:**
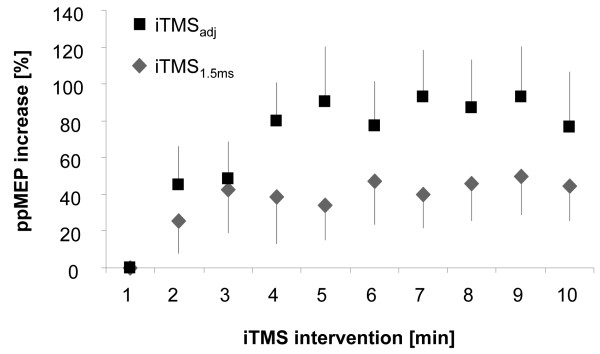
**Comparison between iTMS_1.5 ms _and iTMS_adj_**. Group mean ppMEPs increase [%] during 10 minutes of conventional iTMS_1.5 ms _and iTMS_adj _(n = 13). Please note that there was a significant difference between the amounts of ppMEP facilitation between both interventions. For details see text.

Comparing the effect of iTMS_1.5 ms _with iTMS_adj _(ANOVA_RM_, n = 13) over the left M1 revealed a significant main effect of TIME (1-10 min) (F_(9,108) _= 5.337; p = 0.001) and GROUP (iTMS_1.5 ms _and iTMS_adj_) (F_(1,12) _= 5.412; p = 0.038) while the interaction TIME × GROUP did not reach significance (F_(9,108) _= 1.299; p = 0.246). These results indicate that mean ppMEPs during intervention were significantly higher in iTMS_adj _(mean ppMEPs increase: 76.80 ± 21.81%) as compared to iTMS_1.5 ms _(mean ppMEPs increase: 40.87 ± 19.55%; p = 0.038; Figure 3) while there was no superior effect of iTMS_adj _that develops over time relative to iTMS_1.5 ms_.

**Figure 4:**
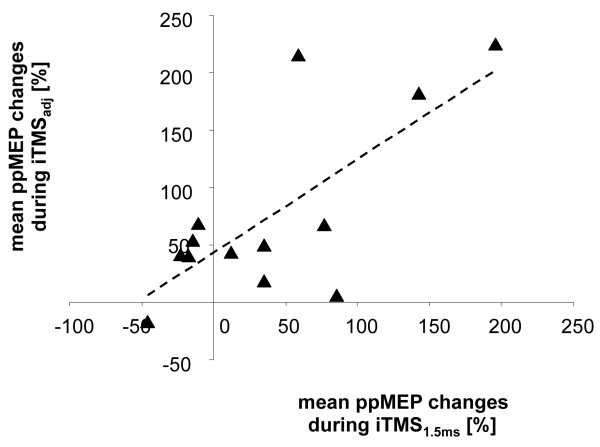
**Correlation analysis**. Linear correlation analysis between the amount of mean ppMEP changes during iTMS_1.5 ms _and iTMS_adj _[%]. Subjects that experienced the most prominent ppMEP changes during iTMS_1.5 ms _also had the largest changes in ppMEP during iTMS_adj _(r = 0.726; p = 0.005). However, those subjects with only little changes after iTMS_1.5 ms _were those that showed strong further facilitation after adjusting for the individual I1-wave peak latency (iTMS_adj_).

In a further step we asked whether those subjects with a large increase in ppMEPs during iTMS_1.5 ms _also showed a comparable change during iTMS_adj_. Indeed, we found a significant positive linear correlation between the amount of changes in I-wave facilitation during iTMS_1.5 ms _and iTMS_adj _(r = 0.726; p = 0.005; see Figure 4). Even more strikingly, in those subjects that showed an overall decrease in ppMEPs during iTMS_1.5 ms _(n = 5), 4 out of 5 subjects showed a clear facilitation in ppMEPs during iTMS_adj_.

## Discussion

In the present study we confirm previously published findings about the efficacy of a conventional iTMS_1.5 ms _protocol applied over M1 to induce significant ppMEP facilitation during intervention [4-6,9]. In comparison to the original report by Thickbroom et al. (2006) [5] the amount of ppMEP facilitation in our study was somewhat weaker, more variable and did not steadily increase, possibly due to the fact that 5 out of 16 subjects tested in our study showed an overall inhibition of ppMEP amplitude during intervention (10 minutes of iTMS_1.5 ms_). The reason behind the lack of response in 5 out of 16 subjects tested (iTMS_1.5 ms_) remains elusive and requires further investigation. However, it might be reasonable to assume that our observation might be related to interindividual differences in the brain-derived neurotrophic factor gene (BDNF) gene. In fact it has been shown that a genetic variation in BDNF can produce significant differences in the after-effects of different rTMS protocols [10]. Apart from genetic factors, other potential determinants such as attention or the synaptic history might explain the lack of effect in some of our subjects.

Nevertheless, here we provide novel evidence that adjusting for the individual I1-wave peak latency resulted in an overall stronger effect during stimulation as compared to iTMS_1.5 ms_. These results indicate that the efficiency of iTMS for the induction of I-wave facilitation strongly depends on the individual choice of the IPI and that optimization of the iTMS protocol might increase the effect of this novel non-invasive brain stimulation technique. However, it is important to keep in mind that the rate of change in ppMEPs over time during stimulation does not seem to depend on the individual IPI chosen for iTMS_adj _since we were not able to identify a significant TIME × GROUP effect.

The rationale behind the efficacy of iTMS is that it presumably targets interneuronal networks involved in the generation of high-frequency descending volleys in the corticospinal tract also known as I-waves [5]. These I-waves have a periodicity of approximately 1.5 ms [11,12] and might result from a transsynaptic activation of corticospinal neurons via excitatory interneurons [1]. With an almost synchronous arrival of the second TMS pulse applied 1.5 ms after the first TMS pulse, the resulting ppMEP facilitation can be interpreted in the framework of Hebbian-like plasticity [13]. However, several other studies indicate that the I-wave periodicity might slightly vary between subjects [7,14]. Indeed, Cash et al. (2009) found after recording I-wave facilitation curves in healthy subjects an average I1-wave peak latency ranging around 1.3 ms [6]. Even though the interval of 1.5 ms used in conventional iTMS protocols might still be "close enough" to induce some Hebbian-like plasticity, in those subjects in whom the second TMS pulse arrives asynchronously with respect to the I-waves generated by the first TMS pulse, the effect may not be optimized. Consequently, we hypothesize that the stronger effect of iTMS_adj _on ppMEP facilitation can be at least partially explained by an optimized "synchronicity" of descending volleys evoked by repetitive paired-pulse TMS. One clear limitation of the present study is that we did not measure corticospinal excitability changes within the left M1 after both interventions (iTMS_1.5 ms _and iTMS_adj_). Therefore, the question whether the stronger facilitation of ppMEPs during iTMS_adj _also translates into a greater increase in corticospinal excitability within the stimulated cortical area (M1) after intervention as compared to iTMS_1.5 ms _remains open and still needs further investigation. However, in a recent report by Fitzgerald and colleagues (2007) they were not able to find any significant change in cortical excitability after 15 minutes of conventional iTMS_1.5 ms _[15]. Therefore, it seems unlikely that 10 minutes of either iTMS_1.5 ms _or iTMS_adj _result in any significant excitability change within the stimulated cortical area. Future studies with longer stimulation durations of iTMS_adj _might be performed to answer this important question.

Another limitation of the present study might be that we only choose a limited number of IPIs for recording the I1-wave peak latency in each subject (1.2 - 1.8 ms). Indeed, Figure 2 indicates that most of the subjects had an I1-wave peak latency of about 1.2 ms. Therefore, it would be reasonable to assume that at least in some subjects the I1-wave peak latency might be even smaller, for example ranging around 1.1 or even 1 ms. Taking this limitation in the estimation of the I1-wave peak latency into account, this would potentially lead to different results as compared to our present study approach.

What are the advantages of using iTMS protocols in comparison to other non-invasive brain stimulation techniques and why is it so important to perform parameter optimization of the conventional iTMS_1.5 ms _protocol?

Previous work indicates that iTMS_1.5 ms _is capable of inducing a substantial increase in corticospinal excitability (around 400% of MEP size, [5]) after stimulation which in comparison to other non-invasive brain stimulation protocols seems to be superior. Another advantage of iTMS is the use of low-frequency TMS pulses (0.2 Hz) which makes the stimulation comfortable for the subjects. Therefore conventional iTMS_1.5 ms _or iTMS_adj _(as shown in the present study) might be a powerful non-invasive technique to overcome the limited and sometimes variable effects of conventional non-invasive brain stimulation protocols. Even though we did not check for iTMS-induced after-effects in the present study one potential limitation of iTMS interventions (until now) are the relatively short-lasting after-effects of only approx. 10 minutes (for review see [15]). For example, the application of theta burst stimulation (TBS) for just 190 seconds has been shown to increase corticospinal excitability for at least 20 minutes [16]. Nevertheless, it remains an open question whether parameter optimization such as adjusting for the individual I-wave latency might result in longer lasting after-effects as compared to iTMS_1.5 ms_.

In summary, our study provides novel evidence that parameter optimization is capable of boosting the efficacy of conventional iTMS_1.5 ms _protocols during intervention. This in turn might have a significant impact on the therapeutic potential of iTMS in neurological disorders.

## Methods

In the present study, we included a total number of 16 healthy subjects between 18 and 35 years of age (5 females). Subjects gave written informed consent to participate in the experiment according to the declaration of Helsinki and the ethics committee of the University of Leipzig approved the study. Prior to participation, all subjects underwent a comprehensive neurological examination. They were not taking any medication. Subjects that did not meet the protocol criteria and/or had contraindications for the study procedures were excluded from participation. According to the Oldfield questionnaire for the assessment of handedness [17], all subjects were right-handed. In a cross-over design, subjects were randomly allocated on the first day of the experiment into one of the following intervention groups: (A) conventional iTMS with an IPI of 1.5 ms (iTMS_1.5 ms_) and (B) iTMS with an individually adjusted IPI (iTMS_adj_). Both interventions were applied on separate days (reversed order, one week apart) in order to control for potential carry-over effects. Since the aim of the present study was to compare the effects of iTMS_1.5 ms _with iTMS_adj_, a total number of three subjects were excluded from data analysis since they showed an individual I-wave periodicity of 1.5 ms.

During the experiment, subjects were seated in an armchair with both arms relaxed and were instructed to keep their eyes open. Surface electromyogram (EMG) was recorded from the first dorsal interosseous (FDI) muscle of the right hand using surface Ag/AgCl electrodes in a bipolar montage. The signal was amplified using an EMG device (D360 8-channel amplifier, Digitimer Ltd, Welwyn Garden City, Hertfordshire, UK) with band pass filtering between 50 and 2000 Hz. The signal was digitized at a frequency of 5000 Hz (CED Power 1401, Cambridge Electronic Design, Cambridge, UK) and fed off-line to a data acquisition system (Signal Version 4.02 for Windows, Cambridge Electronic Design, Cambridge, UK) for further analysis. The absence of voluntary contraction during TMS was monitored online by visual inspection of the EMG signal and off-line by inspection of each individual trace. Trials with background EMG were excluded from the analysis.

TMS stimuli were delivered using a Magstim 200 (Magstim Co., Whitland, South West Wales, UK) through a figure-of-eight 70 mm coil. Initially, the position of the coil was identified over the motor cortex with the handle of the coil pointing posterolaterally with a 45° angle to the sagittal plane to elicit the largest and most consistent MEP amplitude in the right FDI hand muscle. This position was marked (= motor hotspot) [18] using a frameless stereotaxic neuronavigation system (Brainsight™, Montreal, Canada) and monitored online throughout the experiment to exclude any movement of the coil during the stimulation period. The motor hotspot of the FDI muscle representation was identified as the scalp position at which single TMS pulses at slightly suprathreshold intensity induced the most consistent MEP amplitudes in the relaxed muscle.

Resting motor threshold (RMT) over the left M1 was defined as the lowest intensity capable of evoking 5 out of 10 MEPs with amplitudes of at least 50 μV in the relaxed contralateral FDI muscle [18]. TMS pulses were delivered at 0.2 Hz to the left M1 motor hotspot, a rate that does not affect cortical excitability at rest [19].

iTMS_1.5 ms _was applied over the left M1 as previously described [5]. In brief, for iTMS_1.5 ms _paired-pulse TMS pulses of equal strength were delivered at an IPI of 1.5 ms and a repetition rate of 0.2 Hz for 10 minutes, resulting in a total number of 120 paired-pulse TMS stimuli. Stimulus intensity was initially adjusted to generate a MEP of approximately 0.5 - 1.0 mV resulting from paired-pulse TMS and was kept constant throughout the intervention. The reason for choosing an iTMS protocol (10 minutes) shorter than the "standard" 30 min iTMS intervention was to reduce the effective time of the study protocol.

For iTMS_adj_, we first measured the individual I-wave peak latency as indexed by the first I-wave peak (I1-wave peak latency) non-invasively by means of paired-pulse TMS as previously described [6,7]. Stimulus intensity was kept constant for each TMS pulse, and adjusted to generate a MEP of approximately 1 mV when delivered alone, and no more than 4 mV when delivered as a 1.5 ms TMS pulse pair. A total number of 7 IPIs ranging between 1.2 and 1.8 ms in 0.1 ms steps (1.2, 1.3, 1.4, 1.5, 1.6, 1.7 and 1.8 ms) were recorded and compared to a single pulse TMS condition where test MEPs of approximately 1 mV amplitude were elicited. For each IPI and test condition a total number of 10 TMS pulses were recorded. The I1-wave peak latency for each subject was determined as the IPI in which the largest MEP facilitation out of all IPIs could be identified. Subsequently, iTMS was applied with the respective IPI (iTMS_adj_) while keeping the remaining parameters identical to the iTMS_1.5 ms _condition. Therefore, the only difference between iTMS_1.5 ms _and iTMS_adj _was the IPI between both interventions.

Data were analyzed using PASW for Windows version 18. In order to identify ppMEP facilitation in both iTMS interventions separately, we first performed a repeated measures ANOVA (ANOVA_RM_) with factor TIME (1-10 min). Mean amplitudes of ppMEPs obtained in each minute (total of 10 ppMEPs per minute) of both interventions were calculated for each subject and expressed as a percentage of the mean data for the first minute [5]. In order to compare the effect of iTMS_1.5 ms _and iTMS_adj _on ppMEP facilitation we performed an ANOVA_RM_, if necessary with Greenhouse-Geisser sphericity correction, with factor TIME (1-10 min) and GROUP (iTMS_1.5 ms _and iTMS_adj_). Correlation analysis was performed using a linear Pearson correlation coefficient. Group mean data is presented as mean ± standard error of the mean (s.e.m.).

## Authors' contributions

SS, AV and PR conceived the study. SS performed the experiment and was supported by HV, VC, MT and PR. SS and PR analyzed the data. All authors discussed the results. SS and PR wrote the paper. All authors read and approved the final manuscript.
